# Gram-Scale Synthesis of Bimetallic ZIFs and Their Thermal Conversion to Nanoporous Carbon Materials

**DOI:** 10.3390/nano9121796

**Published:** 2019-12-17

**Authors:** Freddy Marpaung, Teahoon Park, Minjun Kim, Jin Woo Yi, Jianjian Lin, Jie Wang, Bing Ding, Hyunsoo Lim, Konstantin Konstantinov, Yusuke Yamauchi, Jongbeom Na, Jeonghun Kim

**Affiliations:** 1 Australian Institute for Innovative Materials (AIIM), University of Wollongong, Squires Way, North Wollongong, NSW 2500, Australia; freddy.marpaung@bppt.go.id (F.M.); konstan@uow.edu.au (K.K.); 2 Key Laboratory of Eco-Chemical Engineering, College of Chemistry and Molecular Engineering, Qingdao University of Science and Technology, Qingdao 266042, China; jianjian_lin@qust.edu.cn (J.L.); y.yamauchi@uq.edu.au (Y.Y.); 3 Carbon Composite Department, Composites Research Division, Korea Institute of Materials Science (KIMS), 797, Changwon-daero, Seongsan-gu, Changwon-si 51508, Gyeongsangnam-do, Korea; thpark@kims.re.kr (T.P.); yjw0628@kims.re.kr (J.W.Y.); 4 School of Chemical Engineering and Australian Institute for Bioengineering and Nanotechnology (AIBN), The University of Queensland, Brisbane, QLD 4072, Australia; minjun.kim@uq.edu.au (M.K.); h.lim@uq.edu.au (H.L.); 5 International Research Center for Materials Nanoarchitechtonics (WPI-MANA), National Institute for Materials Science (NIMS), 1-1 Namiki, Tsukuba, Ibaraki 305–0044, Japan; WANG.Jie@nims.go.jp (J.W.); bingding@nuaa.edu.cn (B.D.); 6 Department of Chemistry, Kookmin University, 77 Jeongneung ro, Seongbuk gu, Seoul 02707, Korea

**Keywords:** hybrid MOFs, nanoporous carbon, microporous, activated carbon, capacitor

## Abstract

The hybrid metal-organic frameworks (MOFs) with different Zn^2+^/Co^2+^ ratios are synthesized at room temperature with deionized water as the solvent. This use of deionized water can increase the yield of hybrid MOFs (up to 65–70%). After the pyrolysis, the obtained nanoporous carbons (NPCs) show a decrease in the surface area, in which the highest surface area is 655 m^2^ g^−1^. The as-prepared NPCs are subjected to activation with KOH in order to increase their surface area and convert cobalt nanoparticles (Co NPs) to Co oxides. These activated carbons are applied to electrical double-layer capacitors (EDLCs) and pseudocapacitors due to the presence of CoO and Co_3_O_4_ nanoparticles in the carbon framework, leading to significantly enhanced specific capacitance as compared to that of pristine NPCs. This synthetic method can be utilized in future research to enhance pseudocapacitance further while maintaining the maximum surface area of the carbon materials.

## 1. Introduction

Continuous consumption of fossil fuels has imposed a negative effect on the environment by accelerating global warming emissions and air pollution. To relieve these environmental stresses, researchers have put great efforts into developing renewable energy sources, including solar energy, wind power energy, geothermal energy, etc. [1,2,3,4,5,6,7]. Such energy supply, however, is not yet sufficiently stable, and its efficiency is greatly influenced by external conditions. Therefore, it is essential to establish an effective and efficient energy storage system to allow renewable energy to be stored for later use [8,9,10]. Together with secondary batteries, supercapacitors are one of the most commonly utilized types of energy storage devices [11,12,13]. Supercapacitors are classified into electrical double-layer capacitors (EDLCs) and pseudocapacitors based on their distinctive charge storage mechanisms. In EDLCs, electrical charge is stored via physical adsorption/desorption of electrolyte ions on the interfaces of electrode/electrolyte through electrostatic attractions. Pseudocapacitors, on the other hand, exploit a surface redox reaction to store electrical charge. EDLCs have gained significant attention due to their outstanding electrochemical advantages, including high power densities, fast charge/discharge as well as superior cyclability. However, their poor energy density, low voltage per cell, high rate of self-discharge, and costly production remain as major challenges to achieve more advanced and functional supercapacitors.

Metal-organic frameworks (MOFs) are formed via coordination bonding between metal ions and organic ligands, which often results in highly ordered porous structures [14,15,16]. Due to the rich content of organic ligands, serving as carbon-sources, MOFs can be utilized as promising carbon precursors to give rise to highly functional carbon materials [17,18,19,20,21]. MOF-derived carbons have been investigated for a broad range of electrochemical applications due to their intrinsic characteristics such as high surface area, controlled chemical functionality, and highly uniform pore sizes. A class of MOFs known as zeolitic imidazolate frameworks (ZIFs) recently have been synthesized in various organic solvents such as methanol, N,N-dimethylformamide (DMF), N,N-diethylformamide (DEF), and dimethyl sulfoxide (DMSO) [22,23,24,25]. Recently, Kim et al. have successfully synthesized hybrid ZIFs with dodecahedral shape by using methanol as the organic solvent [26]. The obtained hybrid ZIFs in methanol, however, show a relatively low yield. Moreover, methanol is toxic, highly flammable, and costly, like other organic solvents commonly used for MOF synthesis.

Interestingly, the electrochemical properties of MOF-derived carbons can be improved by incorporating metal, metal oxide, or metal sulfide nanoparticles onto the carbon surface [27]. For example, Kim et al. successfully synthesized hybrid Co/Zn-ZIF-derived nanoporous carbons (NPCs), which were functionalized with a high content of Co_3_O_4_ nanoparticles (NPs) after oxidation [28]. Due to the presence of both EDLC and pseudocapacitance, the obtained NPCs show a high specific capacitance of up to 545 F g^−1^ and good electrochemical stability with nearly 70% capacity retention after 10,000 charge-discharge cycles. Such ZIF-8/ZIF-67 hybrid ZIF-derived carbons are reported to exhibit a higher graphitic degree than ZIF-8 derived carbons and to allow functionalization of carbon with Co species. In this study, we synthesize hybrid ZIF-8/ZIF-67-derived carbons with different Zn to Co ratios and activate the carbons with KOH [28]. The KOH activation of hybrid ZIF-derived carbons allows avoiding the large loss of specific surface area while obtaining CoO and Co_3_O_4_ in the carbon matrix. The existing CoO and Co_3_O_4_ act as the surface redox-active sites, giving rise to both pseudocapacitance and EDLC.

## 2. Methods

### 2.1. Synthesis of Hybrid MOFs Having Bimetallic Ions (Zn^2+^/Co^2+^) with Different Molar Ratios

Hybrid ZIFs were synthesized in three different Zn^2+^ to Co^2+^ molar ratios (3:1, 1:1, and 1:3). For MOF-3:1, 1.125 g of Zn(CH_3_CO_2_)_2_·2H_2_O and 0.425 g of Co(CH_3_CO_2_)_2_·4H_2_O were dissolved in 50 mL deionized water. For MOF-1:1, 0.750 g of Zn(CH_3_CO_2_)_2_·2H_2_O and 0.850 g of Co(CH_3_CO_2_)_2_·4H_2_O were dissolved in 50 mL deionized water. For MOF-1:3, 0.375 g of Zn(CH_3_CO_2_)_2_·2H_2_O and 1.276 g of Co(CH_3_CO_2_)_2_·4H_2_O were dissolved in 50 mL deionized water. 5.6 g of 2-methyl imidazole was dissolved in separate 50 mL deionized water and then mixed with each sample metal solution. After stirring for 10 min, the resulting solution was kept at room temperature for 24 h. As-synthesized hybrid ZIFs were thoroughly washed several times with deionized water and dried at 60 °C. The resulting MOFs were denoted as MOF-3:1, MOF-1:1, and MOF-1:3, respectively. The same experimental procedures were followed with methanol as a solvent.

### 2.2. Carbonization of Hybrid ZIFs

The ZIF powders were carbonized at a temperature of 800 °C for 3 h under nitrogen with a heating rate of 5 °C min^−1^. The obtained carbon particles were washed several times with deionized water to increase the pH close to 7.0. The resulting NPCs were denoted as NPC-3:1, NPC-1:1, and NPC-1:3, respectively.

### 2.3. Activation of NPCs

The NPCs were immersed in concentrated KOH solution and dried at room temperature prior to the pyrolysis process. The obtained powders were pyrolyzed at 800 °C under nitrogen with a heating rate of 5 °C min^−1^, and then they were washed with 1M HCl solution. The obtained samples were denoted as AC-3:1, AC-1:1, and AC-1:3, respectively.

### 2.4. Characterization

Field emission-scanning electron microscopy (FE-SEM) was operated with a JSM-7100F microscope (JEOL Ltd., Tokyo, Japan) at 10.0 kV. Transmission electron microscopy (TEM) and scanning TEM (STEM) were operated at 100 kV by HT7700 (Hitachi Ltd., Tokyo, Japan). Powder X-ray diffraction (PXRD, D8 Advance, Bruker, Billerica, MA, USA) was used to study crystal structures of materials with Cu–Kα radiation at 40 kV and 40 mA. The elemental composition and the electric structure were investigated by Kratos Axis Ultra photoelectron spectrometer (Kratos Analytical Inc., Manchester, UK) using mono Al Kα (1486.6 eV) X-rays. N_2_ adsorption-desorption isotherms were measured by Quadrasorb SI (Quantachrome Instrument, Boynton Beach, FL, USA). The specific surface area and pore size distribution were obtained by using the Brunauer-Emmett-Teller (BET) method and the non-localized density functional theory (NLDFT) method, respectively.

### 2.5. Electrochemical Measurement

Electrochemical measurements were taken with a CHI660E electrochemical workstation (CH Instruments, Inc., Austin, TX, USA) at room temperature. Three-electrode cell with Pt counter electrode, Hg/HgO reference electrode, and 6 M KOH electrolyte was used. The working electrode solution was prepared with the as-prepared NPC and AC samples, carbon black and PVDF at 8:1:1 weight ratio in 100 µL of N-methyl-2-pyrrolidone (NMP). The solution was thoroughly mixed by ultrasonication before loading onto 1 cm × 1 cm graphite electrode. The working electrode was then vacuum dried at 60 °C overnight. Cyclic voltammetry was measured at multiple scan rates of 1, 5, 10, 20, 50, 100, 200, 300, and 500 mV s^−1^.

## 3. Results and Discussion

**Figure 1:**
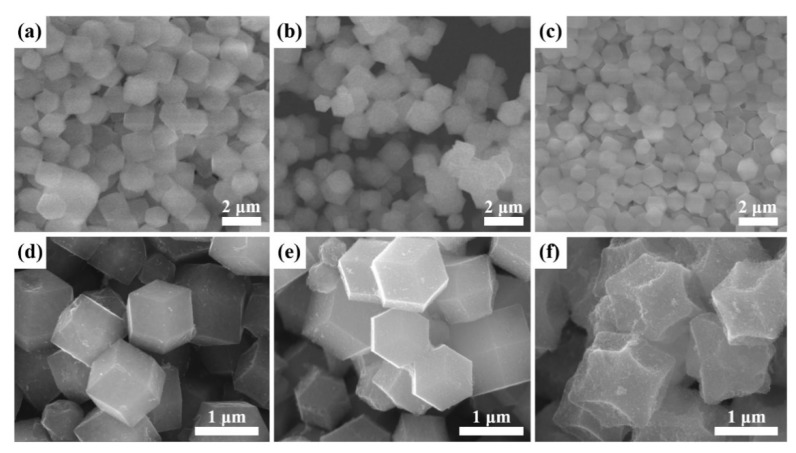
Scanning electron microscopy (SEM) images of (**a**) metal-organic frameworks (MOF)-3:1, (**b**) MOF-1:1, (**c**) MOF-1:3, (**d**) nanoporous carbons (NPC)-3:1, (**e**) NPC-1:1 and (**f**) NPC-1:3, respectively.

**Figure 2:**
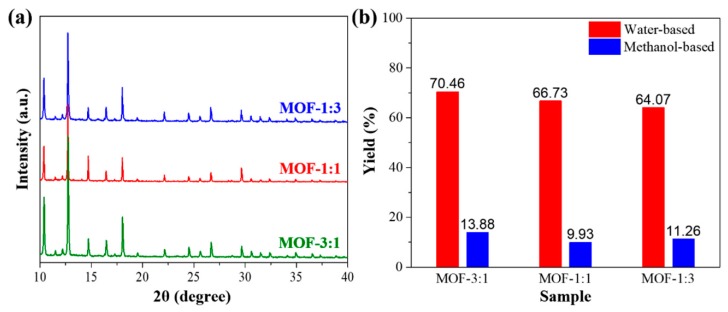
(**a**) Wide-angle X-ray diffraction (XRD) of MOF-3:1, MOF-1:1, and MOF-1:3. (**b**) Comparison of yield (%) of MOF-3:1, MOF-1:1, and MOF-1: between water-based synthesis and methanol-based synthesis. The yield is calculated based on the total mole of both Zn and Co metal species.

SEM images of hybrid bimetallic MOFs (MOF-3:1, MOF-1:1, and MOF-1:3) show typical dodecahedral shape with particle sizes varying from 1 to 3 µm (Figure 1a–c). Wide-angle XRD patterns of MOFs synthesized in deionized water show a highly similar crystal structure with MOFs prepared in methanol while the yield (%) of hybrid MOFs in the water-based synthesis can be increased to more than five times higher than that in methanol-based synthesis (Figure 2). Hence, the water-based synthetic strategy can vastly improve the final yield of hybrid MOFs without changing the MOF crystal structure. According to nitrogen adsorption-desorption isotherms, three types of hybrid MOFs show type I isotherm with a rapid uptake of N_2_ at low relative pressure, clearly indicating a characteristic of microporous materials. Table 1 summarizes the specific surface area (SSA) and the pore volume of hybrid MOF-3:1, MOF-1:1, and MOF-1:3, respectively.

**Figure 3:**
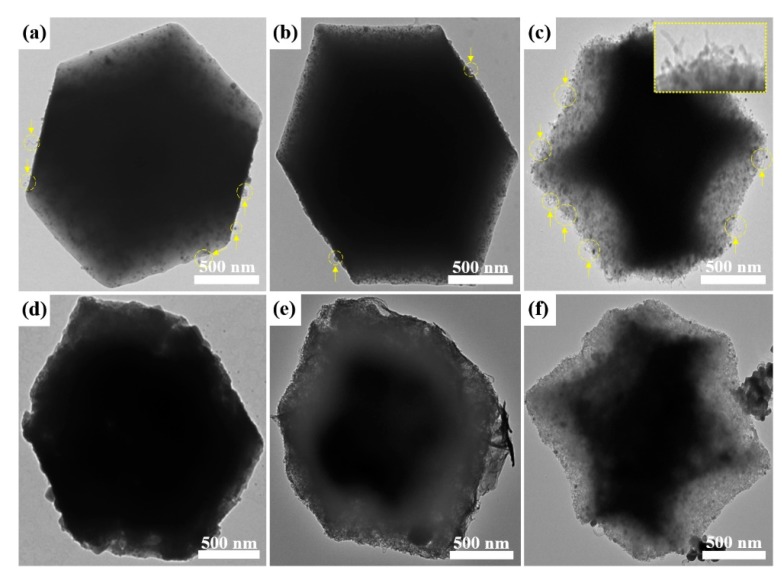
Transmission electron microscopy (TEM) images of (**a**) NPC-3:1, (**b**) NPC-1:1, (**c**) NPC-1:3, (**d**) activated carbon (AC)-3:1, (**e**) AC-1:1, and (**f**) AC-1:3, respectively. Yellow arrows in (**a**–**c**) indicate the presence of carbon nanotubes (CNTs). Inset figure in (**c**) shows the enlarged image of CNTs on the surface of the AC-1:3.

The NPC particles after carbonization maintain a typical morphology with dodecahedral structures (Figure 1d–f), similar to the original morphology of the hybrid MOFs (Figure 1). It is observed that NPC-3:1 and NPC-1:1 show a smooth and well-defined surface morphology, while NPC-1:3 shows a certain level of deformation. Interestingly, TEM images of NPCs show the formation of carbon nanotubes (CNTs) on the surface after the pyrolysis, as indicated by arrows (Figure 3a–c). The Co NPs on the carbon matrix are known to act as catalytic sites for the growth of graphitic CNTs [26,29]. It is important to note that the extent of CNT formation on NPCs is greater for NPCs with the higher molar ratio of Co of their parent MOFs.

**Figure 4:**
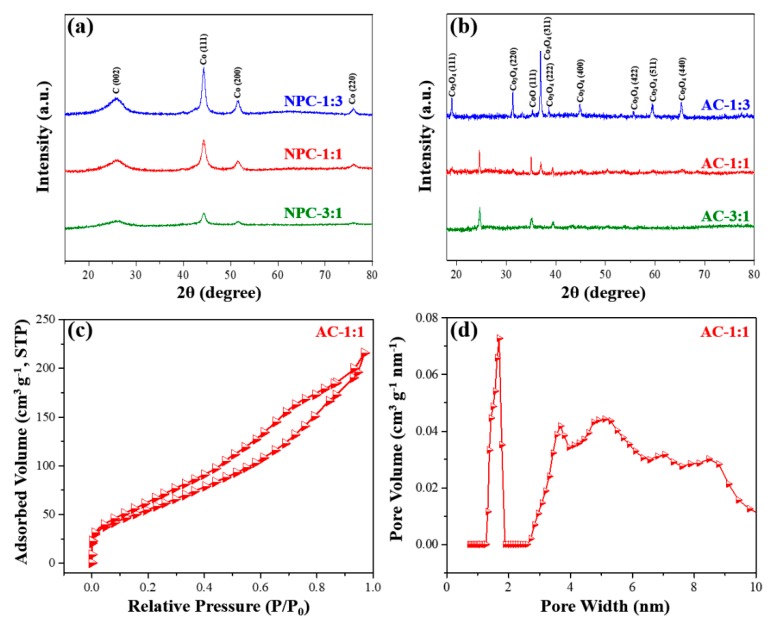
Wide-angle XRD for (**a**) NPC-1:3, NPC-1:1, and NPC-3:1 (before activation) and (**b**) AC-1:3, AC-1:1, AC-3:1 (after activation). The peaks for cobalt and cobalt oxides are denoted. (**c**) N_2_ adsorption-desorption isotherms for AC-1:1 and (**d**) pore size distribution of AC-1:1.

The wide-angle XRD patterns of NPC samples are shown in Figure 4a. Two typical carbon peaks are observable at around 26°, which corresponds to the (002) peak of graphitic carbon. When the Co content is higher in the starting MOFs, the (111), (200), and (220) peaks are more obvious. The average size of the Co NPs was calculated from the broadening of peaks using the Scherrer equation. The average sizes of the Co NPs are around 4.0, 4.6, and 6.3 nm, for NPC-3:1, NPC-1:1, and NPC-1:3, respectively. From the nitrogen adsorption-desorption isotherms for NPC-3:1, NPC-1:1, and NPC-1:3, the SSA of the NPCs is 655 m^2^ g^−1^ (NPC-3:1), 551 m^2^ g^−1^ (NPC-1:1) and 413 m^2^ g^−1^ (NPC-1:3), while the pore volume is 0.238 cm^3^ g^−1^ (NPC-3:1), 0.388 cm^3^ g^−1^ (NPC-1:1), and 0.579 cm^3^ g^−1^ (NPC-1:3). These surface areas are relatively lower in comparison with ZIF-8-derived carbon [30,31]. The results show that the SSA is decreased with the increase in cobalt content.

**Figure 5:**
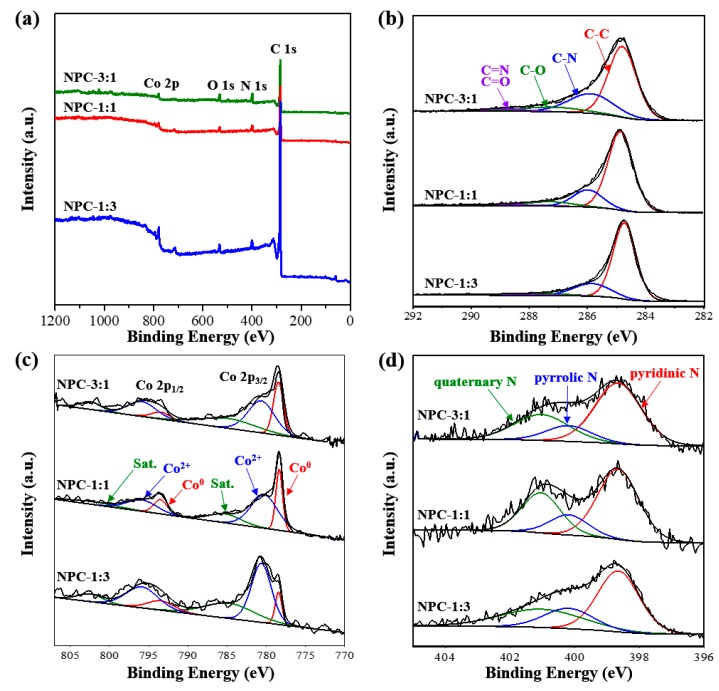
XPS spectra of NPCs. (**a**) Survey X-ray photoelectron spectroscopy (XPS) spectra, (**b**) C 1s XPS spectra, (**c**) Co 2p XPS spectra, and (**d**) O 1s XPS spectra, respectively.

To understand the surface chemical composition and valence states of as-synthesized NPCs, we carefully analyzed the XPS data. As shown in Figure 5, it is revealed that all the samples include carbon, cobalt, oxygen, and nitrogen. Most of the Zn content can be removed/evaporated at high-temperature carbonization process [30,31]. From the XPS analysis (Table 2), the proportion of carbon from NPC-3:1 to NPC-1:3 were calculated to be 83.1 at.% (NPC-3:1), 89.5 at.% (NPC-1:1), 93.5 at.% (NPC-1:3), respectively. From Figure 5c, it is found that the Co metal surface is slightly oxidized, which is not detected by the above wide-angle XRD measurement (Figure 4a).

**Figure 6:**
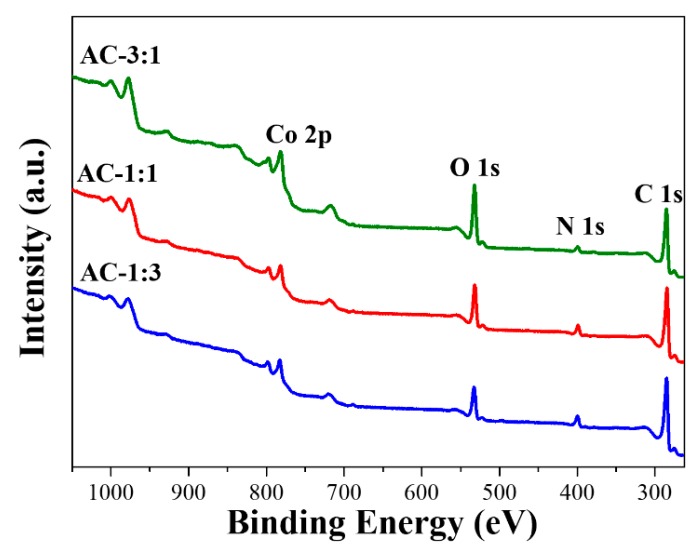
XPS survey for AC-3:1, AC-1:1, and AC-1:3, respectively.

Figure 3d–f clearly shows the effect of KOH activation on the morphology of NPCs. Basically, the original NPC shapes remain, but the surface of ACs tend to become much rougher compared to their respective NPCs due to chemical etching by KOH. Wide-angle XRD patterns of AC samples show co-existence of CoO and Co_3_O_4_ as well as Co (Figure 4b), indicating that the original Co is partially or fully oxidized to CoO and Co_3_O_4_ during the KOH activation process. The SSA and pore volume for ACs are characterized by N_2_ adsorption-desorption isotherms (Figure 4c–d and Figure S1). According to Table 1, three AC samples show to reduce the SSAs compared to their NPC samples. This observation can be partly attributed to the increased weight density of ACs due to the formation of Co_3_O_4_ NPs and the reduction of carbon amount. The element compositional ratios of ACs from survey XPS (Figure 6 and Table 2) show higher compositional content of Co with respect to that of C in comparison to the NPCs.

**Figure 7:**
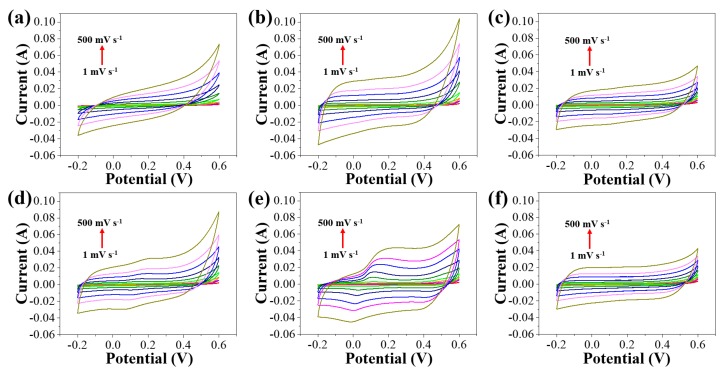
CVs for (**a**) NPC-3:1, (**b**) NPC-1:1, (**c**) NPC-1:3, (**d**) AC-3:1, (**e**) AC-1:1, and (**f**) AC-1:3.

**Figure 8:**
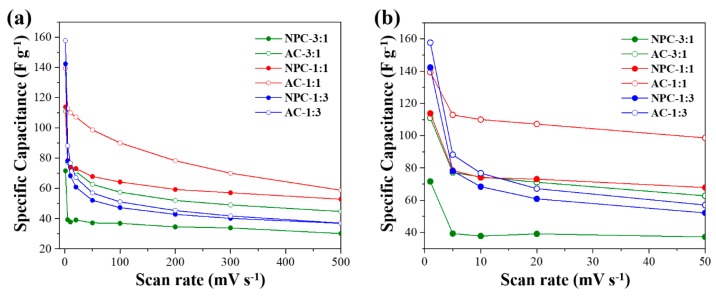
Specific capacitance at different scan rates at (**a**) 1, 5, 10, 20, 50, 100, 200, 300, and 500 mV s^−1^, (**b**) 1, 5, 10, 20, and 50 mV s^−1^.

The electrochemical performance of the as-prepared NPC and AC samples were evaluated from cyclic voltammetry (CV), as shown in Figure 7. The CV of all AC samples clearly shows the co-existence of EDLC and pseudocapacitance, whereas only EDLC is mainly observed in the CV of NPC samples. According to wide-angle XRD patterns (Figure 4b), the redox activity of the ACs can be attributed to the presence of CoO or Co_3_O_4_ NPs, which are formed via partial or full oxidation of Co NPs in the AC samples. The two cobalt oxide species are well-known for their distinctive pseudocapacitance [32]. However, there is no observable redox activity in AC-1:3, possibly due to the encapsulation of Co species within the carbon framework. This is further evidenced by our XPS elemental analysis showing lower Co content in AC-1:3 as compared to AC-3:1 and AC-1:1 (Table 2) [29]. Consequently, each AC sample exhibits a significantly enhanced specific capacitance at all scan rates (1, 5, 10, 20, 50, 100, 200, 300, 500 mV s^−1^) as compared to their pristine NPC (Figure 8). After activation, the specific capacitance is clearly increased. Among samples prepared with different Zn^2+^ to Co^2+^ molar ratio, AC-1:1 exhibits the most superior specific capacitance over a broad range of scan rates. Especially at a higher scan rate, AC-1:1 exhibits the greatest specific capacitance among the three AC samples, indicating that AC-1:1 is highly electrochemically stable at a rapid scan rate (Figure 8). This is largely due to the optimized compositional ratio of Co and O of AC-1:1 originating from CoO and Co_3_O_4_ NPs on the surface, thus giving rise to greater pseudocapacitance together with EDLC.

## 4. Conclusions

Hybrid MOFs with different Zn^2+^/Co^2+^ ratios are synthesized at room temperature using water-based synthesis. The use of deionized water as a solvent allows a significantly increased yield of hybrid MOFs, where the maximum yield reaches around 65–70%. The resulting hybrid MOFs show type-I isotherm, which is typical for microporous material with high SSA. After the pyrolysis process, the obtained NPCs show a decrease in surface area, in which the highest surface area is 655 m^2^ g^−1^ for NPC-3:1. It is also found that the CNTs content is significantly increased by increasing the Co content. The as-prepared NPCs are subsequently subjected to activation with KOH in order to increase their surface area and convert Co NPs to Co oxides. The resulting activated carbons (AC-1:3, 1:1 and 3:1) exhibit both EDLC and pseudocapacitance due to the presence of CoO and Co_3_O_4_ nanoparticles in the carbon framework, leading to significantly enhanced specific capacitance as compared to their pristine NPCs. Among the three AC samples, AC-1:1 shows stable electrochemical performance as its specific capacitance outcompetes that of other samples at all scan rates. The water-based synthetic method reported in this study is highly efficient and environmental-friendly. Furthermore, oxidation of Co NPs in hybrid MOFs using KOH activation process can be utilized in future research to maximize pseudocapacitance further while maintaining the maximum surface area of the carbon materials. Recently, many types of functional MOF-derived materials with different compositions have been reported by several groups [33,34,35,36]. Therefore, the optimal activation method reported here will be useful for further upgrading the material performance in the future.

## Figures and Tables

**Table 1:** Summary of surface area and pore volume of ACs.

Sample	Surface Area (m^2^ g^−1^)	Pore Volume (cm^3^ g^−1^)
MOF-3:1	1393	0.126
MOF-1:1	1414	0.124
MOF-1:3	1417	0.103
NPC-3:1	655	0.238
NPC-1:1	551	0.388
NPC-1:3	413	0.579
AC-3:1	423	0.416
AC-1:1	205	0.335
AC-1:3	224	0.453

**Table 2:** XPS elemental analysis data of various NPC and AC samples.

Elemental Analysis (at.%)					
Sample	Zn	Co	C	N	O
NPC-3:1	0.37	0.70	83.1	10.8	5.08
NPC-1:1	-	1.11	89.5	6.42	3.00
NPC-1:3	-	0.94	93.5	3.70	1.84
AC-3:1	0.05	2.50	84.03	3.92	9.50
AC-1:1	-	5.28	78.63	2.36	13.73
AC-1:3	0.01	3.35	80.30	6.05	10.28

## Supplementary Materials

The following are available online at https://www.mdpi.com/2079-4991/9/12/1796/s1, Figure S1: N_2_ adsorption-desorption isotherms and pore size distribution for AC-1:3 and AC-3:1.

## References

[1] Liu M., Johnston M.B., Snaith H.J. (2013). Efficient planar heterojunction perovskite solar cells by vapour deposition. Nature.

[2] Na J., Kim Y., Park C., Kim E. (2015). Multi-layering of a nanopatterned TiO_2_ layer for highly efficient solid-state solar cells. NPG Asia Mater..

[3] Staffell I., Pfenninger S. (2016). Using bias-corrected reanalysis to simulate current and future wind power output. Energy.

[4] Kumar Y., Ringenberg J., Depuru S.S., Devabhaktuni V.K., Lee J.W., Nikolaidis E., Andersen B., Afjeh A. (2016). Wind energy: Trends and enabling technologies. Renew. Sustain. Energy Rev..

[5] Zheng B., Xu J., Ni T., Li M. (2015). Geothermal energy utilization trends from a technological paradigm perspective. Renew. Energy.

[6] Li K., Bian H., Liu C., Zhang D., Yang Y. (2015). Comparison of geothermal with solar and wind power generation systems. Renew. Sustain. Energy Rev..

[7] Na J., Kim J., Park C., Kim E. (2014). TiO_2_ nanoparticulate-wire hybrids for highly efficient solid-state dye-sensitized solar cells using SSP-PEDOTs. Rsc Adv..

[8] Pramanik M., Tsujimoto Y., Malgras V., Dou S.X., Kim J.H., Yamauchi Y. (2015). Mesoporous iron phosphonate electrodes with crystalline frameworks for lithium-ion batteries. Chem. Mater..

[9] Hwang S.M., Lim Y.-G., Kim J.-G., Heo Y.-U., Lim J.H., Yamauchi Y., Park M.-S., Kim Y.-J., Dou S.X., Kim J.H. (2014). A case study on fibrous porous SnO_2_ anode for robust, high-capacity lithium-ion batteries. Nano Energy.

[10] Xue H., Zhao J., Tang J., Gong H., He P., Zhou H., Yamauchi Y., He J. (2016). High-Loading Nano-SnO_2_ Encapsulated in situ in Three-Dimensional Rigid Porous Carbon for Superior Lithium-Ion Batteries. Chem. Eur. J..

[11] Huang H.S., Chang K.H., Suzuki N., Yamauchi Y., Hu C.C., Wu K.C.W. (2013). Evaporation-Induced Coating of Hydrous Ruthenium Oxide on Mesoporous Silica Nanoparticles to Develop High-Performance Supercapacitors. Small.

[12] Bastakoti B.P., Huang H.-S., Chen L.-C., Wu K.C.-W., Yamauchi Y. (2012). Block copolymer assisted synthesis of porous α-Ni(OH)_2_ microflowers with high surface areas as electrochemical pseudocapacitor materials. Chem. Commun..

[13] Makino S., Yamauchi Y., Sugimoto W. (2013). Synthesis of electro-deposited ordered mesoporous RuO_x_ using lyotropic liquid crystal and application toward micro-supercapacitors. J. Power Sour..

[14] Eddaoudi M., Kim J., Rosi N., Vodak D., Wachter J., O’Keeffe M., Yaghi O.M. (2002). Systematic design of pore size and functionality in isoreticular MOFs and their application in methane storage. Science.

[15] Stock N., Biswas S. (2011). Synthesis of metal-organic frameworks (MOFs): Routes to various MOF topologies, morphologies, and composites. Chem. Rev..

[16] Maurin G., Serre C., Cooper A., Férey G. (2017). The new age of MOFs and of their porous-related solids. Chem. Soc. Rev..

[17] Tang J., Yamauchi Y. (2016). Carbon materials: MOF morphologies in control. Nat. Chem..

[18] Zhang W., Jiang X., Zhao Y., Carné-Sánchez A., Malgras V., Kim J., Kim J.H., Wang S., Liu J., Jiang J.-S. (2017). Hollow carbon nanobubbles: Monocrystalline MOF nanobubbles and their pyrolysis. Chem. Sci..

[19] Wang C., Kaneti Y.V., Bando Y., Lin J., Li J., Yamauchi Y. (2018). Metal-organic framework-derived one-dimensional porous or hollow carbon-based nanofibers for energy storage and conversion. Mater. Horiz..

[20] Torad N.L., Li Y., Ishihara S., Ariga K., Kamachi Y., Lian H.-Y., Hamoudi H., Sakka Y., Chaikittisilp W., Wu K.C.-W. (2014). MOF-derived nanoporous carbon as intracellular drug delivery carriers. Chem. Lett..

[21] Zhang W., Jiang X., Wang X., Kaneti Y.V., Chen Y., Liu J., Jiang J.S., Yamauchi Y., Hu M. (2017). Spontaneous Weaving of Graphitic Carbon Networks Synthesized by Pyrolysis of ZIF-67 Crystals. Angew. Chem. Int. Ed..

[22] Young C., Salunkhe R.R., Tang J., Hu C.-C., Shahabuddin M., Yanmaz E., Hossain M.S.A., Kim J.H., Yamauchi Y. (2016). Zeolitic imidazolate framework (ZIF-8) derived nanoporous carbon: The effect of carbonization temperature on the supercapacitor performance in an aqueous electrolyte. Phys. Chem. Chem. Phys..

[23] Salunkhe R.R., Young C., Tang J., Takei T., Ide Y., Kobayashi N., Yamauchi Y. (2016). A high-performance supercapacitor cell based on ZIF-8-derived nanoporous carbon using an organic electrolyte. Chem. Commun..

[24] Park K.S., Ni Z., Côté A.P., Choi J.Y., Huang R., Uribe-Romo F.J., Chae H.K., O’Keeffe M., Yaghi O.M. (2006). Exceptional chemical and thermal stability of zeolitic imidazolate frameworks. Proc. Natl. Acad. Sci. USA.

[25] Ameloot R., Gobechiya E., Uji-i H., Martens J.A., Hofkens J., Alaerts L., Sels B.F., De Vos D.E. (2010). Direct patterning of oriented metal–organic framework crystals via control over crystallization kinetics in clear precursor solutions. Adv. Mater..

[26] Kim J., Young C., Lee J., Park M.-S., Shahabuddin M., Yamauchi Y., Kim J.H. (2016). CNTs grown on nanoporous carbon from zeolitic imidazolate frameworks for supercapacitors. Chem. Commun..

[27] Kaneti Y.V., Tang J., Salunkhe R.R., Jiang X., Yu A., Wu K.C.W., Yamauchi Y. (2017). Nanoarchitectured design of porous materials and nanocomposites from metal-organic frameworks. Adv. Mater..

[28] Kim J., Young C., Lee J., Heo Y.-U., Park M.-S., Hossain M.S.A., Yamauchi Y., Kim J.H. (2017). Nanoarchitecture of MOF-derived nanoporous functional composites for hybrid supercapacitors. J. Mater. Chem. A.

[29] Torad N.L., Hu M., Ishihara S., Sukegawa H., Belik A.A., Imura M., Ariga K., Sakka Y., Yamauchi Y. (2014). Direct synthesis of MOF-derived nanoporous carbon with magnetic Co nanoparticles toward efficient water treatment. Small.

[30] Torad N.L., Hu M., Kamachi Y., Takai K., Imura M., Naito M., Yamauchi Y. (2013). Facile synthesis of nanoporous carbons with controlled particle sizes by direct carbonization of monodispersed ZIF-8 crystals. Chem. Commun..

[31] Chaikittisilp W., Hu M., Wang H., Huang H.-S., Fujita T., Wu K.C.-W., Chen L.-C., Yamauchi Y., Ariga K. (2012). Nanoporous carbons through direct carbonization of a zeolitic imidazolate framework for supercapacitor electrodes. Chem. Commun..

[32] Paulraj A.R., Kiros Y. (2018). La_0.1_Ca_0.9_MnO_3_/Co_3_O_4_ for oxygen reduction and evolution reactions (ORER) in alkaline electrolyte. J. Solid State Electr..

[33] Tatykayev B., Donat F., Alem H., Balan L., Medjahdi G., Uralbekov B., Schneider R. (2017). Synthesis of core/shell ZnO/rGO nanoparticles by calcination of ZIF-8/rGO composites and their photocatalytic activity. ACS Omega.

[34] Xiong Y., Xu W., Zhu Z., Xue Q., Lu W., Ding D., Zhu L. (2017). ZIF-derived porous ZnO-Co_3_O_4_ hollow polyhedrons heterostructure with highly enhanced ethanol detection performance. Sens. Actuators B Chem..

[35] Zhuang G., Gao Y., Zhou X., Tao X., Luo J., Gao Y., Yan Y., Gao P., Zhong X., Wang J. (2017). ZIF-67/COF-derived highly dispersed Co_3_O_4_/N-doped porous carbon with excellent performance for oxygen evolution reaction and Li-ion batteries. Chem. Eng. J..

[36] Tuan D.D., Kun-Yi Andrew L. (2018). ZIF-67-derived Co_3_O_4_ rhombic dodecahedron as an efficient non-noble-metal catalyst for hydrogen generation from borohydride hydrolysis. J. Taiwan Inst. Chem. Eng..

