# Supplementary material for: Gram-Scale Synthesis of Bimetallic ZIFs and Their Thermal Conversion to Nanoporous Carbon Materials
Source: Nanomaterials (Basel). 2019 Dec 17;9(12):1796. doi: 10.3390/nano9121796 (PMC6955874; doi:10.3390/nano9121796)
Supplement: Supplementary file 1 [file nanomaterials-09-01796-s001.pdf]

Supplementary Materials

Gram-Scale Synthesis of Bimetallic ZIFs and Their Thermal Conversion to Nanoporous Carbon Materials

Freddy Marpaung [1,2,†], Teahoon Park [3,†], Minjun Kim [4,†], Jin Woo Yi [3], Jianjian Lin [2], Jie Wang [5], Bing Ding [5], Hyunsoo Lim [4], Konstantin Konstantinov [1], Yusuke Yamauchi [2,4,5], Jongbeom Na [2,4,5,*] and Jeonghun Kim [2,6,*]

1 Australian Institute for Innovative Materials (AIIM), University of Wollongong, Squires Way, North Wollongong, NSW 2500, Australia; freddy.marpaung@bppt.go.id (F.M.); konstan@uow.edu.au (K.K.)
2 Key Laboratory of Eco-Chemical Engineering, College of Chemistry and Molecular Engineering, Qingdao University of Science and Technology, Qingdao 266042, China; jianjian_lin@qust.edu.cn (J.L.); y.yamauchi@uq.edu.au (Y.Y.)
3 Carbon Composite Department, Composites Research Division, Korea Institute of Materials Science (KIMS), 797, Changwon-daero, Seongsan-gu, Changwon-si 51508, Gyeongsangnam-do, Korea; thpark@kims.re.kr (T.P.); yjw0628@kims.re.kr (J.W.Y.)
4 School of Chemical Engineering and Australian Institute for Bioengineering and Nanotechnology (AIBN), The University of Queensland, Brisbane, QLD 4072, Australia; minjun.kim@uq.edu.au (M.K.); h.lim@uq.edu.au (H.L.)
5 International Research Center for Materials Nanoarchitechtonics (WPI-MANA), National Institute for Materials Science (NIMS), 1-1 Namiki, Tsukuba, Ibaraki 305–0044, Japan; WANG.Jie@nims.go.jp (J.W.); bingding@nuaa.edu.cn (B.D.)
6 Department of Chemistry, Kookmin University, 77 Jeongneung ro, Seongbuk gu, Seoul 02707, Korea
* Correspondence: j.na@uq.edu.au (J.N.); jeonghunkim@kookmin.ac.kr (J.K.)
† These authors contributed equally to this work.

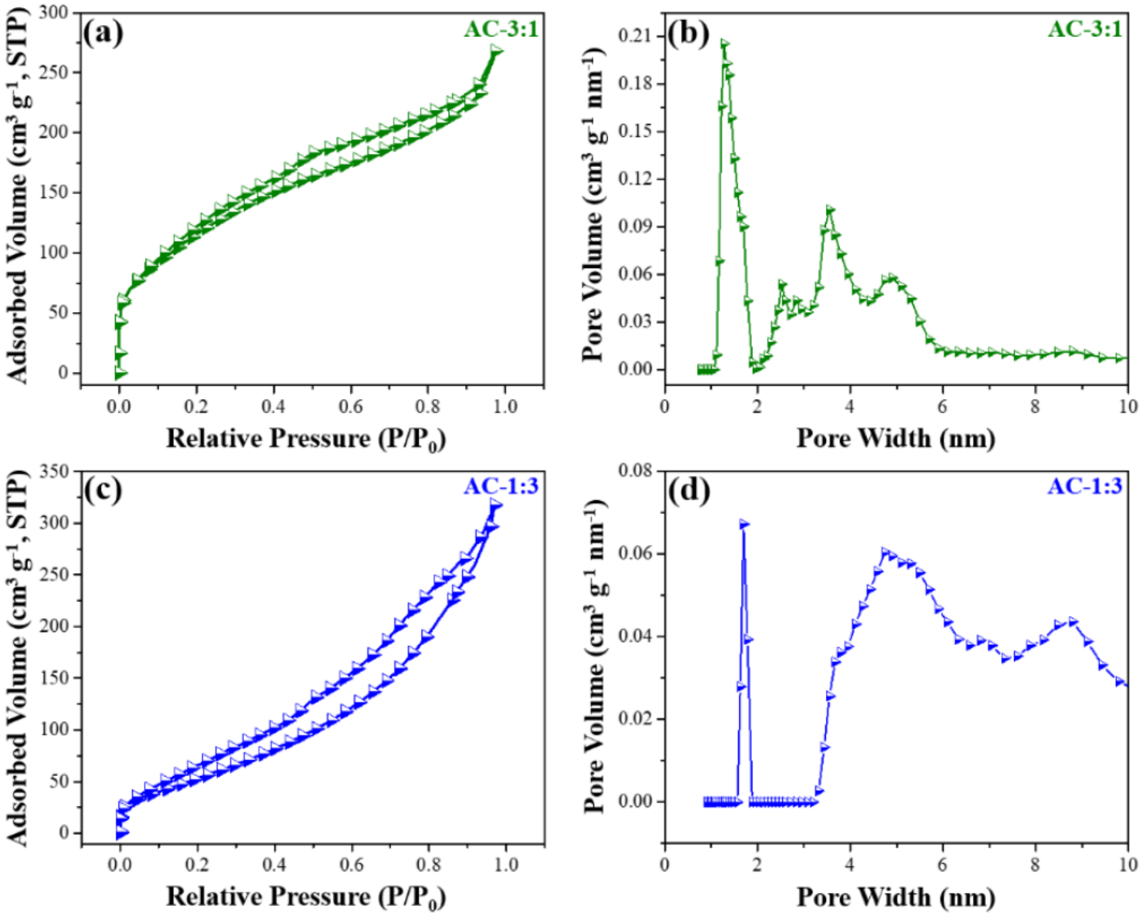

Figure S1. (a) N_2 adsorption-desorption isotherms for AC-1:3, (d) pore size distribution of AC-1:3, (c) N_2 adsorption-desorption isotherms for AC-3:1 and (d) pore size distribution of AC-3:1.
